# Advances in the Repurposing and Blood–Brain Barrier Penetrance of Drugs in Pediatric Brain Tumors

**DOI:** 10.3390/cancers17030439

**Published:** 2025-01-27

**Authors:** Julian S. Rechberger, Stephanie A. Toll, Subhasree Biswas, Hyo Bin You, William D. Chow, Nicholas Kendall, Pournima Navalkele, Soumen Khatua

**Affiliations:** 1 Department of Neurologic Surgery, Mayo Clinic, Rochester, MN 55905, USA; rechberger.julian@mayo.edu; 2 Children’s Hospital of Michigan, Central Michigan University School of Medicine, Saginaw, MI 48602, USA; toll1s@cmich.edu; 3 Bronglais General Hospital, Caradog Road, Aberystwyth SY23 1ER, Wales, UK; subhabis@gmail.com; 4 Mayo Clinic Alix School of Medicine, Mayo Clinic College of Medicine and Science, Rochester, MN 55905, USA; you.hyobin@mayo.edu (H.B.Y.); chow.william@mayo.edu (W.D.C.); 5 School of Medicine, University of South Dakota Sanford, Vermillion, SD 57069, USA; kendall.nicholas@mayo.edu; 6 Division of Oncology, Children’s Hospital of Orange County, Orange, CA 92868, USA; pournima.navalkele@choc.org; 7 Department of Pediatric Hematology/Oncology, Mayo Clinic College of Medicine and Science, Rochester, MN 55905, USA

**Keywords:** pediatric brain tumors, blood–brain barrier, drug delivery, drug repurposing, clinical trials

## Abstract

Central nervous system (CNS) tumors are the leading cause of cancer-related mortality in children, with prognosis remaining dismal for some of these malignancies. Though the past two decades have seen advancements in surgery, radiation, and targeted therapy, major unresolved hurdles continue to undermine the therapeutic efficacy. These include challenges in suboptimal drug delivery through the blood–brain barrier (BBB), marked intra-tumoral molecular heterogeneity, and the elusive tumor microenvironment. Drug repurposing or re-tasking FDA-approved drugs with evidence of penetration into the CNS, using newer methods of intracranial drug delivery facilitating optimal drug exposure, has been an area of intense research. This could be a valuable tool, as most of these agents have already gone through the lengthy process of drug development and the evaluation of safety risks and the optimal pharmacokinetic profile. They can now be used and tested in clinics with an accelerated and different approach. Conclusions: The next-generation therapeutic strategy should prioritize repurposing oncologic and non-oncologic drugs that have been used for other indication, and have demonstrated robust preclinical activity against pediatric brain tumors. In combination with novel drug delivery techniques, these drugs could hold significant therapeutic promise in pediatric neurooncology.

## Simple Summary

Pediatric brain tumors remain a leading cause of cancer-related death in children. Even with advances in the multimodality management of these malignancies, the outcome remains poor in many cases. The development of new drugs has been frustratingly slow, due to the cost and lengthy steps needed for new drug development. To expedite this, drug repurposing is an attractive approach, as it evaluates approved drugs, which have already gone through the safety, pharmacological, and regulatory validation prior to it being driven to clinics. This provides hope for these children who harbor such formidable tumors.

## 1. Introduction

Tumors of the central nervous system (CNS) are the leading cause of cancer-related deaths among children and adolescents [1,2]. The past decade has yielded significant advances in the molecular genomics of these tumors, yet this knowledge has not translated into robust improved outcomes, with a marginal increase of ~1.0% in the overall survival (OS) from the 1970s to the early 2000s [3]. Though the rate of oncology drug growth is rapidly improving, pediatric-specific CNS tumor drug development has unfortunately remained slow, with only ten new Food-and-Drug-Administration (FDA)-approved drugs entering the market since 1953 [4].

Even with the advancements in the multimodal therapies for pediatric CNS tumors, which include safe maximal surgical resection, chemotherapy (including targeted therapy, stem cell transplant), and irradiation therapy, unpredictable relapses and elusive resistance mechanisms continue to undermine the therapeutic efficacy. These tumors are markedly more heterogeneous in their molecular, histological, epidemiological, and prognostic classification than their adult counterparts, making similar treatment inferences across all ages challenging [5]. Pediatric low-grade glioma (LGG) is a prime example of this, with treatment varying widely from the standard of care in adults.

LGGs are the most common CNS tumors in the pediatric population, treated with conventional chemotherapy, and irradiation is usually reserved for those tumors with inoperable or sub-totally resectable disease. Unfortunately, both of these strategies are fraught with toxicities. The last decade has witnessed significant advances in identifying novel molecular signatures and oncogenic drivers of these tumors, paving the way to develop targeted therapy against these malignancies. The most common molecular aberration in these tumors is activation of the mitogen-activated protein kinase (MAPK) pathway. This has led to promising studies targeting the key genomic alterations in MEK (mitogen-activated extracellular signal-regulated kinase) and BRAF in this pathway. However, paradoxical MAPK pathway activation with BRAF inhibitor monotherapy resulted in the development of early relapse. This led to the development of a combinatorial therapy with a repurposing approach, using previously tested drugs, the BRAF along with MEK inhibitors, Dabrafenib and Trametinib, respectively. A synergistic benefit with prolonged remission was noted with this therapy [6,7].

Various subtypes of pediatric HGG are now identified with continuing molecular revelations, as defined in the WHO 2021 classification of CNS tumors [8,9]. The poorest prognostic subtype is H3 K27M-altered tumors, in which the OS is dismal at <10% [10,11]. Conversely, infant-type hemispheric glioma, with NTRK, ROS1, ALK, or MET aberrations, have improved outcomes, with 5-year overall survival ranging from 25 to 53.8% [12].

Pediatric embryonal tumors harbor unique genetic and epigenetic signatures, resulting in the need for stratified treatment strategies. Current regimens depend on the age and biological subtype, with young children receiving irradiation-sparing therapy post-surgically and older children receiving craniospinal irradiation followed by maintenance chemotherapy. The OS is dependent on the molecular subgroup, with TP53-mutated sonic hedgehog (SHH) and Group 3 (non-SHH and non-WNT) bearing the worst outcomes and WN- mutated and TP53-wild-type SHH demonstrating near universal survival rates [13,14,15,16].

The heterogeneous tumor microenvironment (TME) in pediatric CNS tumors plays a crucial role in both disease development and progression precluding therapeutic efficacy. An improved understanding of the cross-talks between the tumor and its microenvironment, escape mechanisms of the tumor from the host-protective immune system, and underpinnings in the development of therapeutic resistance, have now helped in profiling effective therapies, bypassing these encumbrances.

Unlike adults, CNS tumors in children have a low mutational burden, providing fewer antigens to be attacked by the host immune system, hindering the development of targeted therapies. These tumors are immunologically “cold”, characterized by the low expression of immunogenic markers and a paucity of cytokine immune infiltrates, thus being poorly reactive to the host immune defense system. On the contrary, the increased presence of immune-regulatory cells, like myeloid-derived suppressor cells (MDSCs) and T regulatory cells (T_reg_s), undermines the desired inflammatory response to targeted therapy. Furthermore, immunosuppressive molecules in the TME, like tumor-associated macrophages (TAM) and TGFβ, hinder the immune response to treatment [17].

A large methylation study evaluated the immune phenotype of more than 6000 pediatric CNS samples, which showed three immune clusters that were statistically significant, correlating with molecular subgroups/subtypes, mutations, and prognosis [18]. Some of the clinically relevant insights are discussed below.

The number of immune infiltrates is higher in LGG compared to HGG, with most infiltrating cells being macrophages and T cells [19]. Pediatric LGG and HGG have mildly increased CD8+ T cell infiltration, while diffuse midline glioma (H3K27 altered) does not [17]. Though these higher-grade gliomas have increased immunosuppressive TAMs (macrophage type 2, also known as CD163 cells), this immune infiltration does not uniformly correlate with poor prognosis, unlike in adult glioblastoma [18,20]. Medulloblastoma (MB), the most common malignant pediatric brain tumor, also has a cold TME and the lowest immune infiltrates when compared to other pediatric CNS tumors [21]. The immunophenotyping analysis of a large cohort of pediatric MB patients revealed the predictive capacity of the immune cell infiltrate population towards survival outcomes in the molecular subtypes of MB [22,23]. Understanding these immunological phenotypes in different pediatric CNS tumors is now providing clues to define optimal therapeutic regimens.

Though these newer biological insights are promising, we still face significant hurdles in optimizing the drug concentration at tumor sites [24]. This is largely attributed to the BBB, a unique complex barrier consisting of a near impenetrable system, made of endothelial cells, astrocytes, pericytes, neurons, and an extracellular matrix [25]. While this tightly orchestrated interplay is critical for self-protection, it results in a markedly decreased permeability for drug delivery. The phenotypic heterogeneity of the BBB across molecular subtypes of primary pediatric CNS tumors makes it even more challenging to determine the best drug-delivery method.

Even with exciting research advances in the molecular basis of pediatric neuro-oncogenesis, current treatment outcomes have remained prognostically static, along with a miniscule pace of profiling new drugs against pediatric CNS tumors. A major challenge is the protracted time and enormous cost of developing a new drug. A newer expedited attractive approach is drug repositioning, using approved or investigational drugs with already established pharmacokinetics, pharmacodynamics, and safety profiles, facilitating quicker patient access to newer treatment options.

The following section provides updates on the repurposing of drugs (oncologic and non-oncologic) for pediatric brain tumors and reviews various methods of intracranial drug delivery, which mitigate the concerning systemic toxicity, augment the drug concentration into the tumors, and improve therapeutic efficacy.

## 2. Repurposing of Drugs for Brain Tumors

The last few decades have witnessed an outpouring of new biological data including druggable targets, in pediatric brain tumors. However, translational of new drugs to the clinics has been painstakingly slow [26,27]. Major obstacles of bringing a new drug from bench to bedside, are the protracted time of nearly 13 years, cost of 2–3 billion dollars and a low success rate of approximately 12% [28]. After a drug of interest is identified, it goes through pharmacokinetic (PK) and pharmacodynamic (PD) evaluation, involving in vitro and in vivo studies and assessment of toxicity. It then proceeds for regulatory approval, prior to moving into a phase I study in humans. This phase evaluates feasibility, safety, and toxicity profile, along with a tolerated dose level finding. If successful, it will move into a phase II and III study after IRB or FDA approval. Even with all these efforts, only one in 5000–10,000 prospective anticancer agents receive FDA approval, and only 5% of oncology drugs entering Phase I clinical trials are ultimately approved [29]. This is an arduous and lengthy process, resulting in significant delay in drug availability for cancer patients, with a recent trend in lack of interest by the pharmaceutical industry in developing a new drug. Thus, there is a dire need to develop a therapeutic innovation of a quicker, effective, and safer method of drug development for cancer patients, overcoming the decades of bottleneck of profiling new cancer therapeutics.

A promising next-generation therapeutic strategy of drug repurposing, also known as repositioning or redirecting, is now actively pursued in pediatric neurooncology. This novel concept allows drugs or investigational agents, which are FDA approved and used for other indications (oncologic or non-oncologic), including widely available generic medications, to be assessed for repurposing. The advantage of this approach is the drugs of interest have already gone through a rigorous process of preclinical studies, including its evaluation of PK, PD, drug interaction parameters, toxicity profile, and phase I studies, for a different noncancer morbidity or another type of malignancy. They can now be translated into clinical use without the need for structural or chemical modification. This concept proved successful as witnessed by the expedited approval of drugs during the 2020 COVID-19 pandemic [30,31]. Renewed interest by the industry in developing cancer drugs using this strategy has been kindled, with the potential of decreased time and cost along with need for much less regulatory barriers [32].

Similarities in cancer specific biological pathways or mechanisms of action (MOA), have enabled drugs used for different malignancy or non-oncologic conditions, to be expeditiously redirected for brain tumors. Other avenues for repurposing are identifying effective combinations of drugs with synergy targeting several signaling pathways, helping to overcome progression of adaptive therapy resistance. Another attractive method is redirecting drugs to the CNS, using novel drug delivery methods, undermining disconcerting toxicities seen with systemic therapy [33,34]. In addition, these repurposed drugs used alone or in combination, have the potential of targeting multiple unrelated oncogenic pathways driving tumorigenesis, including undruggable targets, a distinct advantage over single molecularly targeted therapy [35].

Identifying a suitable compound for repurposing in pediatric brain tumors, must have a strong biological and clinical rationale based on unmet need in this vulnerable population. Then the drug of interest will have to fulfill these criteria, before being approved for clinical use. They must have a favorable profile of BBB penetration, established safety in infants and children, proven efficacy in preclinical models of tumors of interest, and pharmacokinetic properties that allow therapeutically effective concentrations at the tumor site. If evaluated for a combinatorial therapy, synergy between the drugs must be demonstrated against the target tumor. Finally, FDA approval is needed, as the drug targets children with an orphan disease, prior to moving it into the clinical arena [2,36].

The initial idea of repurposing has been largely opportunistic and serendipitous, long known since World War II, when the deadly mustard gas was redirected, to develop nitrogen mustard as a chemotherapy for lymphoma. However, it was only recently that the concept of repurposing has been developed strategically with technological advances [37,38]. These include computational and experimental approaches, which help swiftly to identify a suitable medicament of interest for further evaluation. The current computational method based on molecular theory helps to pinpoint a drug by dissecting a large digital record of health information (diagnostic and pathophysiological data) and understanding it at the molecular level. This is complemented by a comprehensive analysis of experimental data, (which enumerates chemical, or protein structure, gene expression and omics profile) of the compound of interest [39]. Different platforms have been implemented for ideal candidate selection and its repurposing. These include the ReDO project, which is a database for candidate selection [40]. DRUGSURV, one of the largest repository of FDA approved and experimental drugs database [41], and Benevolent AI (a leader in advanced artificial intelligence, which generates machine learning based graphical data of drugs and their targets, aiding discovery of rare genomic aberration like the ACVR-mutant DIPG [42], are other pivotal approaches. The last few years have seen a dramatic rise in the use of AI in various steps of anti-cancer drug discovery, resulting in rapid turnover of drug identification, with perfect precision and minimized error of the pharmaceutical industry [43].

Though drug repurposing has gained significant momentum driving optimism in drug discovery and relevant translational research, challenges are concerning. The continuing spurt of biological data, and new molecular insights in various subtypes of pediatric brain tumors, far outpaces the emanating of appropriate preclinical studies including development of animal models to test drugs of interest. Ongoing collaboration between scientists, clinicians, industry and the regulatory teams are often fraught with challenges. Even so, the rapid pace of research and the implementation of repurposing compounds to the treatment of brain cancer, now ushers a new paradigm in cancer therapeutics. Finally, in the words of James Black, Nobel Laureate in physiology and medicine (1988), who once said “the most fruitful basis for the discovery of a new drug is to start with an old drug”, a reminder of the true essence of drug repurposing.

The following tables were generated using a search criterion of key words including childhood brain tumors and repurposed drugs for CNS tumors, retrieved from PubMed and ClinicalTrials.gov data bases. In Table 1, repurposed oncology drugs are presented, while Table 2 provides a comprehensive overview of non-oncologic repurposed drugs. Table 3 highlights outcomes for repurposed oncologic drugs, and Table 4 lists outcomes for repurposed non-oncologic drugs.

## 3. Non-Oncology Drugs Repurposed for Pediatric Brain Tumors

Landmark discoveries in antineoplastic properties of thalidomide in 1990 (which was initially used to treat nausea in pregnancy, and later discontinued for inducing fetal teratogenicity in the 1950’s) and mebendazole (used originally as an antiparasitic), paved the way of intense research evaluating non-oncologic drugs use in cancer [44,45]. To date more than 250 such therapeutics have shown promising anti-cancer properties and are being evaluated for various malignancies [46]. As pediatric brain tumors with their elusive biology continue to undermine therapeutic efficacy, newer approaches are profiled to overcome these impediments. Morbidities like headache, epilepsy, psychiatric illness, and neurodegenerative disease are seen in 20–30% of pediatric brain tumor patients. Drugs used for treating these ailments are now repurposed singly or in combination with conventional therapies, to improve clinical outcome of these malignancies [47,48]. These medications are appealing, as they have shown to cross the BBB in therapeutic concentrations, addressing a major challenge of optimal drug delivery in brain tumors. Moreover, they have antineoplastic properties targeting diverse signaling pathways or oncogenic drivers and would require much less resources and preclinical assessment prior to moving them into the clinical arena.

## 4. The Blood-Brain Barrier

**Figure 1:**
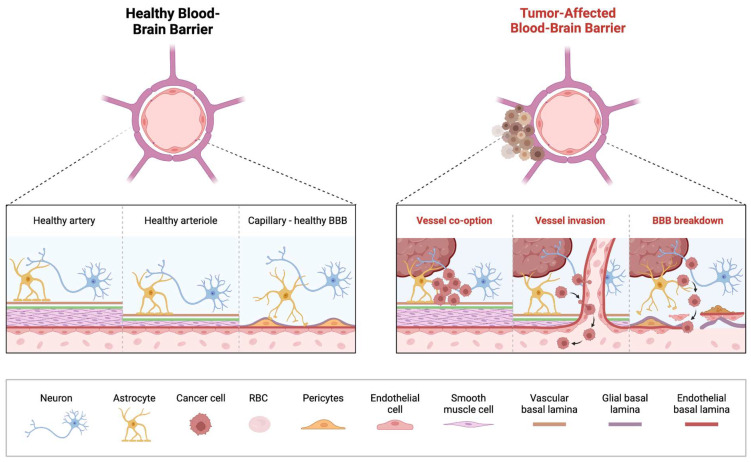
Overview of Blood-Brain Barrier Alterations in Pediatric Brain Tumors. On the (**left**) is an illustration of the healthy BBB with correctly functioning arteries, arterioles and capillaries. On the (**right**) is an illustration of the breakdown of the BBB that takes place in a tumor-affected brain, with vessel co-option by the tumor and subsequent invasion of the vessel resulting in the breakdown of the previously healthy BBB.

The BBB is a critical structure that protects the brain from infection and neurotoxicants. It is composed of various cell types, most prominent of which include astrocytes, pericytes, and perivascular macrophages [49,50] (Figure 1). Together with endothelial cells and tight junctions, the BBB regulates the passage of compounds from the blood into the cerebrospinal fluid (CSF) [51,52,53,54]. Efflux pumps like P-glycoprotein (Pgp), breast cancer resistance protein (BCRP), and multidrug resistance-associated protein 4 (MRP4) actively remove substrates that can bypass the barrier. Nearly 60% of available drugs on the market are substrates of Pgp, making efflux pumps invaluable in preventing toxic accumulation of drugs in the CNS [55]. However, these efflux pumps may be upregulated in gliomas, impeding therapeutic drug delivery [56]. Prior studies have shown that an upregulation of P-gp and BRCP in GBM decreases the delivery of temozolomide (TMZ) [57]. Furthermore, these efflux pumps, specifically P-gp and BCRP, appear to remain active on tumor vasculature despite chemotherapeutic disruption of the BBB; as such, these efflux pumps may continue to decrease therapy efficacy, despite efforts to enhance initial delivery [58]. Previously conducted studies administering palbociclib, gefitinib, sunitinib to P-gp deficient mice have demonstrated markedly elevated levels of drug concentration within the brain as compared to wild-type mice [59,60,61]. However, clinical trials in humans investigating the co-administration of P-gp inhibitors such as elacridar and tariquidar with chemotherapy have been less fruitful, with significant uptake of chemotherapy yet to be demonstrated, along with concerns of toxicity [62,63,64].

Brain tumors contribute to a “leakier” BBB, as cancer cells interfere with the normal function and distribution of cells that compose the BBB [52,65,66,67]. This has been termed the blood-tumor barrier (BTB) wherein the integrity of BBB is compromised, characterized by heterogeneous permeability which can significantly impede delivery of therapeutic agents to the tumor cells [66,67]. Striking differences in the BTB are seen in some of the common malignant pediatric brain tumors, medulloblastoma (MB) and diffuse midline glioma (DMG). The WNT-activated molecular subgroup of MB, displays the most favorable prognosis amongst the four molecular subgroups, and generally responds well to chemotherapy [68,69]. It is believed that the aberrant vasculature network in the WNT-activated MB, enhances the “leaky” BBB, rendering them more susceptible to systemic chemotherapy, as also demonstrated in preclinical models. This contrasts with the formidable Sonic Hedgehog (SHH subgroup) which maintains BBB integrity, hindering access to systemic chemotherapy [68,70,71].

DMG are aggressive tumors, usually located in eloquent areas of the brain, precluding robust surgical intervention. They tend to maintain vascular integrity like the normal brain, preserving the intact nature of the BTB, explaining their mostly non-contrast neuroimaging features, limited drug penetration and poor clinical outcomes [11,71,72,73,74,75]. Histological and preclinical models of DMG showed uninterrupted expression of junctional proteins CLDN5 and CD31, normal expression of the transporter Glut1 and continuing coverage by pericytes, suggesting the maintenance of vascular integrity in presence of tumor cell infiltration [71].

In contrast, little is known about the phenotype of the BTB, BBB proteins and transporter in two other common pediatric malignant brain tumors, ependymoma and the atypical teratoid rhabdoid (ATRT) [24].

These diverse mechanisms impeding adequate BBB penetration of drugs, resulting in suboptimal tumor concentration, warrants the need to profile novel methods of drug delivery to circumvent these obstacles [49,51] (Figure 1).

## 5. Methods of Bypassing the Blood-Brain Barrier

### 5.1. Convection-Enhanced Delivery (CED)

**Figure 2:**
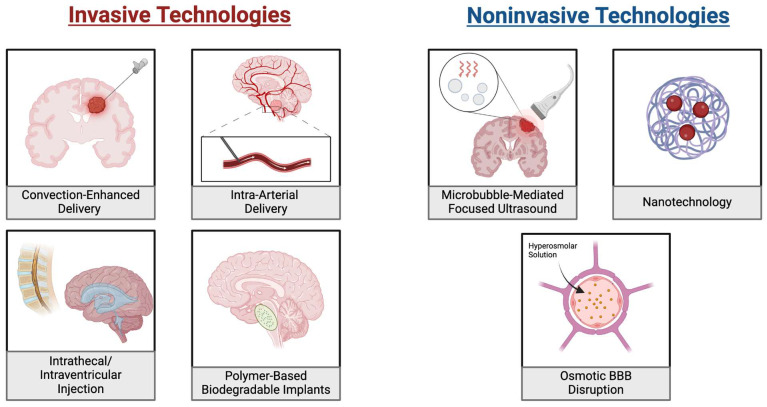
Drug Delivery Approaches for Bypassing the Blood-Brain Barrier in Pediatric Brain Tumors. On the (**left**) are illustrations of four invasive approaches for bypassing the BBB. These include convection-enhanced, intra-arterial and intrathecal/intraventricular methods. Each of these distributes the drug directly at the tumor location or to a system that will reach it without passing the BBB. Additionally, the placement of biodegradable implants is shown where drug-loaded implants are placed near the tumor and gradually release the drug. On the (**right**) are three noninvasive approaches to delivering chemotherapies. These include microbubble mediated focused ultrasound, which uses ultrasound waves to enhance targeted drug delivery and nanotechnology which uses nanoparticles to carry drugs to the tumors. Additionally, osmotic BBB disruption utilizes a hyperosmolar solution to drive osmotic forces which disrupts the integrity of the BBB.

Convection-enhanced Delivery (CED) is an advancement in drug delivery that bypasses the BBB via direct interstitial infusion into a tumor under a pressure gradient [76,77]. It is a direct, targeted delivery mechanism that limits off-target effects, while delivering optimal dosages. The ability to begin therapy with smaller initial drug load, while being able to have a controlled homogenous distribution with minimal systemic absorption, makes CED an attractive option in the pediatric population [77,78,79,80]. Despite challenges (Figure 2), as a growing area of research, CED has significant potential, especially when combined with various synergistic technologies like nanoparticles [81].

Over the last few years, CED has shown to be safe and efficacious in DIPG in vivo models and patients [82,83,84,85,86,87]. Carboplatin has been used systemically for various cancers including brain tumors with significant myelosuppressive toxicity. A study of repurposing carboplatin via intratumoral infusions in pontine lesion, using robot-guided implantation of catheter, demonstrated feasibility of accurately and safely delivering small-diameter catheters [88]. Based on encouraging preclinical data, panobinostat, a pan HDAC inhibitor, demonstrated safety when used orally in DIPG, with myelosuppression as the major toxicity in a phase I trial PBTC-047. Having also demonstrated good penetration through BBB in murine models of DIPG, this drug has now been repurposed using repeated CED infusions of MTX110, a water-soluble nanoparticle of panobinostat into the tumor (NCT03566199) [89].

Irinotecan, an alkylating agent, has been used alone or in combination with other drugs for recurrent HGG in children with variable efficacy and concerns of toxicity [90,91]. To undermine these side effects and increase drug delivery to tumor site, it is now repurposed using CED of a liposomal form of irinotecan injection in DIPG, as a part of ongoing phase I trial (NCT 03086616).

Dasatinib, an oral tyrosine kinase inhibitor, has been used in children with Philadelphia positive leukemia demonstrating improved survival. Recently, it has been used to target PDGFRα in pediatric HGG alone or along with mTOR inhibitors [92,93,94]. Though it demonstrated efficacy, adverse effects were concerning. Ongoing research is now exploring avenues to enhance delivery of dasatinib in brain tumors. ABC transporter inhibition along with dexamethasone, enhances its clinical efficacy when delivered by CED in pontine tumors. This approach demonstrated enhanced tumor cellular apoptosis and survival of the transgenic H3.3K27M mutant murine model of DIPG [82]. Trametinib, a MEK inhibitor, has been used in pediatric LGG and neurofibromatosis (NF) tumors, induced by the activation of the MAPK/ERK pathway, with varied success [95,96]. This drug has been repurposed using a combinatorial approach along with dabrafenib, a BRAF inhibitor, in similar tumors [97]. A promising study showed prolonged survival of intracranial genetic mouse models of DIPG when ZSTK474, a PI3K inhibitor, is used along with trametinib and delivered by CED, warranting further studies using this delivery technique [98].

Radiolabeled monoclonal antibodies (RMA) have been in use along with other drugs and radiation to treat pediatric tumors including leukemias, myelodysplastic syndrome and neuroblastoma [99,100]. Promising preclinical data has paved the way of RMA use through CED in pediatric brain tumors. A first-in-human study of 45 patients with DIPG received theranostic intratumoral CED of ^123^I-Omburtamab. This study demonstrated safety and was able to deliver high radiation doses to DIPG, with a wide safety margin and feasibility of real time tracking of the infused agent [101].

Over the last decade, oncolytic viral therapy (OVT) has garnered much attention as a therapeutic option in pediatric tumors, including brain tumor, osteosarcoma, retinoblastoma, and neuroblastoma. Though these efforts demonstrated safety, ongoing research continues to explore clinical outcomes in repositioning these viral constructs with newer genetic modifications and novel drug delivery methods which would enable less off-target toxicity. This approach is now further evaluated in combination with radiation therapy and immunotherapy to enhance clinical efficacy [102,103]. A recent study of DIPG patients who received intratumoral CED of oncolytic virus DNX-2401 along with radiation therapy, demonstrated safety with some response [86]. A phase 1b trial of twelve pediatric patients with recurrent HGG received intratumoral CED of lerapolturev, a recombinant polio-rhinovirus chimera, which demonstrated safety, affirming its evaluation in a larger cohort of patients (NCT03043391) [104].

Biodegradable implants in which polymers are loaded with various drugs, along with a controlled time release mechanism, can be embedded directly in the tumor bed after surgical resection [105,106]. This delivery mechanism has been explored in adults, but few studies have been done in pediatric brain tumors and they showed minimal efficacy.

Few case reports of efficacy in pediatric HGG were noted, with gliadel wafers used alone or in combination with temozolomide and etoposide [107,108]. Another study of gliadel wafers, used along with low dose oral etoposide, in children with anaplastic ependymoma demonstrated safety but did not show any clinical benefit [109]. The biological difference between adult and pediatric tumors likely limits the use of these in the pediatric population. These limitations include rapid degradation of wafers, unwanted effects of degradation products, short-term drug release kinetics and rapid drug hydrolysis of polymers [105,110]. Ways to undermine these limitations should be pursued in future studies, enabling development of optimal therapy of these implants, in combination with other drugs and radiation.

### 5.2. Intrathecal and Intraventricular Injections

Intrathecal (IT) and intraventricular (IV) injections are the most common ways to deliver drugs directly into the CSF. IT therapy has been used over many years in the treatment of primary and secondary CNS tumors [111,112].

These direct CSF injections have multiple benefits. They can bypass first-pass metabolism and thereby limit systemic toxicity. Being already in the CSF space, they can bypass barriers like BBB and achieve high concentration in the CNS [111,113]. Thus, IT and IV routes of drug delivery are therapeutically promising for lesions that occur in CSF or can spread through CSF such as ependymomas, medulloblastomas, and choroid plexus tumors.

Many drugs which have been evaluated for systemic use, are now being repurposed for delivery via IT or IV route to increase tumor access for increased clinical efficacy with limited side effects of systemic therapy. IT MTX resulted in a significant increase in 5-year PFS and OS and in pediatric patients with high-risk medulloblastoma [112]. Feasibility and safety were demonstrated with fourth ventricular infusions of methotrexate for the treatment of medulloblastoma and ependymoma [114]. Clinical trials of fourth ventricular infusions of methotrexate and etoposide for recurrent posterior fossa tumors in pediatric patients (NCT02905110) and MTX110 for recurrent medulloblastoma (NCT04315064) are ongoing. IT liposomal cytarabine (Ara-C) was well tolerated with clinical efficacy in children with high-risk recurrent cranial embryonal neoplasms [115]. A Phase I pilot study demonstrated safety with repeated fourth ventricular infusions of demethylating agent 5-azacitidine injections in posterior fossa ependymomas with no concerning neurological toxicity [116]. Topotecan, a topoisomerase inhibitor, has been widely used through intravenous or oral route for pediatric brain tumors with variable efficacy and concerning toxicity [117,118]. High-dose chemotherapy with stem cell rescue followed by intrathecal topotecan maintenance therapy could avoid whole brain radiation, showing efficacy in young children with aggressive atypical teratoid rhabdoid tumor (ATRT). This repurposing strategy, though promising, needs to be further evaluated in larger studies [119].

Nivolumab, a checkpoint inhibitor, and 5-azacytidine has been used via oral and systemic route respectively, in various pediatric malignancies. Clinical trials are now repurposing these drugs by IV routes, evaluating for increased tumor concentration, safety and toxicity profile. These studies include fourth ventricular infusions of nivolumab and 5-azacytidine or methotrexate for recurrent ependymoma and medulloblastoma (NCT0646679). A pilot study of 5-azacytidine and trastuzumab (an EZH2 inhibitor) infusions into the fourth ventricle or resection cavity in children and adults with recurrent posterior fossa ependymoma is also ongoing (NCT04958486). A phase 2 study for recurrent medulloblastoma and ependymoma is investigating whether the addition of IV RMA ^131^I-omburtamab to irinotecan, temozolomide, and bevacizumab can improve both the detection and treatment of these tumors. (NCT04743661). A clinical trial of ^131^I-omburtamab for treatment of CNS or leptomeningeal neoplasms in children and young adults is ongoing (NCT05064306).

Chimeric antigen receptor (CAR) T cell therapy has been evaluated against pediatric solid tumors based on promising results in pediatric leukemias. Though safe, it has limited antitumor efficacy due to fewer targetable antigens, hostile TME and suboptimal homing to tumor sites when delivered systemically [120]. Thus, IV or IT delivery of CART cells to brain tumors are now being evaluated for increased tumor concentration and clinical efficacy. Phase I studies in HGG demonstrated feasibility and safety when CAR-T therapy was delivered by IT or IV route [121,122,123,124]. H3K27M DMG express high levels of GD2, which led to a phase I study of initial intravenous infusions of this GD2-CAR T cells in these tumors. Those patients who exhibited clinical benefit, were eligible for subsequent GD2-CAR T cell infusions administered by IV route. Early results underscore the promise of this repositioning therapeutic approach for patients with these formidable tumors [125,126]. Based on increased expression of B7H3 in DIPG, a phase 1 clinical trial is underway to evaluate the safety and efficacy of IV delivery of CAR T cell with IL-7Ra signal, targeting B7H3 in children with DIPG (NCT06221553). A similar trial showed feasibility and safety of IV infusions of B7-H3 CAR T cells for patients with DIPG, with correlative evidence of immune activation, reaffirming efficacy of this novel delivery approach [126]. IV administration of autologous NK cells demonstrated safety and feasibility of its use in recurrent medulloblastoma and ependymoma [127].

### 5.3. Intra-Arterial Delivery (IA)

Cetuximab, an EGFR inhibitor, and bevacizumab, an angiogenesis inhibitor, has been used to treat adults and pediatric CNS tumors using the intravenous route. However, suboptimal tumor drug concentration has undermined clinical efficacy, and toxicity profile were concerning [128,129]. Repositioning these two drugs using super-selective intra-arterial cerebral infusion (SIACI) in pediatric patients with refractory HGG demonstrated safety in a phase 1 trial [130]. Based on this encouraging result, a phase I/II Trial of monthly dosing of SIACI is now enrolling patients with HGG under 22 years of age (NCT05956821).

Improving IA delivery to CNS lesions using transient cerebral hypoperfusion (TCH), has shown some promise in preclinical models, demonstrating enhanced tumor-specific uptake of peptide and liposome-based carrier molecules in glioma and metastatic brain cancer rodent models [131,132,133]. One such study reports glioma uptake increased four-fold with TCH-IA compared to IV with 2.41-fold greater tumor deposition. A recent study in choroid plexus carcinoma genetic mouse model, showed IA administration of melphalan (an alkylating agent) combined with elimusertib (inhibitor of Rad3-related kinase) led to a significant increase in survival and limited toxicity, emphasizing the potential of this delivery technique in pediatric CNS tumors. Both these drugs have been used orally in various cancers with significant toxicity.

Though IA delivery of therapeutics in pediatric CNS tumors has shown some clinical benefits, off-side effects are concerning. These include seizures, high-frequency hearing loss, and irreversible encephalopathy [134,135]. Other side effects mostly seen in adult patients include granulocytopenia, nephrotoxicity, intratumoral hemorrhage, and transient cerebral ischemia, raising concerns for its use in children [136].

### 5.4. Through the Blood Brain Barrier

#### 5.4.1. Focused Ultrasound

Focused ultrasound (FUS) is an emerging technology used for the treatment of adult and pediatric brain tumors, due to its ability to induce diverse biological effects. These include thermal or mechanical tissue ablation, immunomodulation, radiosensitization and BBB disruption [137]. The therapy relies on using IV-administered microbubbles, which generate intravascular mechanical forces in sonicated regions, reflecting transmitted energy onto endothelial cells of local vasculature through oscillation, causing stable cavitation. This allows modulation of the BBB and induces transient separation of tight junctions, facilitating increased drug delivery [138,139,140]. These microbubbles and endothelial cells interactions also result in downregulation of efflux transportation and sonoporation, all of which contribute to enhanced drug delivery into the brain and improved accumulation of targeted agents in the sonicated region [137,141]. This anatomical disruption is transient, and the BBB permeability is restored in 6–24 h, making it an even more appropriate and attractive therapeutic option [141].

Carboplatin, doxorubicin and temozolomide have been used extensively by the intravenous or oral route in pediatric cancers. They are now being evaluated in preclinical models of brain tumors, repurposing their access to tumor site using FUS for increased tumoral concentration and clinical efficacy. Results of these studies are encouraging: a study with doxorubicin demonstrated mean concentration of 5366 ng/g with FUS compared to 251 ng/g while another study with temozolomide achieved 19 ng/mg compared to 6.983 ng/mg [142,143,144]. Based on these promising data, a clinical trial using low-intensity FUS (LIFS) with microbubbles to deliver doxorubicin in DIPG patients is open to accrual. Preliminary results are demonstrating safety (NCT03028246) [145].

DMG remains therapeutically challenging as the BBB remains largely intact and contributes to chemoresistance [146]. Radiotherapy remains the standard of care for these tumors. The potential for synergy of radiation with FUS to enhance clinical efficacy is being assessed in preclinical models [147,148]. A preclinical study using delivery of panobinostat using FUS with microbubbles in a DIPG mouse model demonstrated a three-fold increase in tumoral concentration of the drug (from 61.8 ng/g to 194.3 ng/g), with marked reduction of tumor size and increased survival [149]. These promising results led to an ongoing Phase I clinical study in progressive DMG, evaluating oral panobinostat along with FUS microbubbles and neuro-navigator-controlled sonication (NCT04804709).

A phase I/II trial in DIPG patients is evaluating the feasibility and safety of using an investigational agent, combined with the active ingredient aminolevulinic acid HCl using sonodynamic therapy (SDT) with LIFU. This approach has been shown to sensitize drugs of interest and improve its effect into target tissues. Preliminary results in 10 patients have demonstrated safety [145].

A phase I trial in patients with recurrent GBM is using a skull-implantable ultrasound device that transiently opens the BBB. Using this device with LIFU led to an increase in mean brain parenchymal concentrations of albumin-bound paclitaxel compared to non-sonicated brains from 37.3 nM to 138.6 nM [150]. FUS shows promise in enabling improved drug penetration to tumor sites, even with large therapeutics such as biologics and gene therapies [151,152].

#### 5.4.2. Chemical BBB Disruption

Chemical reagents, such as vasoactive substances, can transiently and reversibly disrupt the tight junctions of endothelial cells lining the cerebral vasculature, improving drug delivery into CNS. These include bradykinin, histamine and arachidonic acid which have been used to increase BBB permeability [148,149,150,151,152,153]. Preclinical studies have shown a bradykinin analog, Lobradimil (RMP-7), induces BBB disruption and facilitates drug uptake into the brain, resulting in a 2.7-fold increase in drug concentration and 74% survival rate at 31 days compared to 37% in the control [154,155]. A phase II study was conducted, using intravenous Lobradimil along with carboplatin in pediatric brain tumors. Though safety was demonstrated, no favorable outcome was achieved, with a suboptimal AUC. A follow up study was planned with a higher dose [156]. In parallel, a phase I study confirmed safety and feasibility of giving this combination daily during radiotherapy to children with brainstem tumors [157].

#### 5.4.3. Osmotic BBB Disruption

Studies have shown promising results of BBB disruptions using hyperosmolar agents like mannitol, lactamide and hypertonic saline. These agents split the tight junctions in the BBB, shrink endothelial cells, and increase the paracellular diffusion to improve delivery of both diagnostic and therapeutic agents to the CNS [158,159,160,161,162,163]. A phase I clinical trial using SIACI delivery of bevacizumab and cetuximab through BBB disruption with mannitol, in pediatric patients with HGG and DMG demonstrated safety and encouraging mean OS of 17.3 months in DIPG patients [130].

Though these modalities are encouraging, risks of seizures, pulmonary edema and renal failure are concerning [160,164,165]. Successful clinical translation to pediatric brain tumors requires understanding safety issues of repeated BBB opening and optimization of treatment that minimizes risk and maximize efficacy of BBB disruption and drug delivery.

#### 5.4.4. Nanotechnology

Nanoparticles (NPs) are surfacing as a promising precision-based tool for diagnosis and a vehicle for drug delivery in brain tumors, due to their increased ability to cross the BBB. Their small size (ranging between 1–1000 nm), easily degradable phenotype and non-toxic profile holds promise in pediatric neurooncology. NPs fall into three major categories: organic (liposomes and polymers), inorganic (metals/metal oxides, ceramics, quantum dots) and carbon-based (fullerenes and nanotubes) [166,167]. They can absorb, entrap, or be modified with various pharmacological agents, facilitating its action as a non-invasive drug delivery vehicle for therapeutics in various cancers [168,169,170,171]. Encapsulating biological agents into various NPs has been exploited to alter the pharmacokinetics (PK) of drugs, improving its drug solubility, transportation through the BBB, biodistribution and delaying drug degradation to optimize clinical efficacy [172,173,174]. This provides an advantage to methods like IT and IV injections, alleviating barriers to use such as rapid clearance from CSF [175] and neurological toxicities including cytokine release syndromes [176].

Panobinostat has been used for the treatment of MB and DMG via oral or CED route. However, due to its poor water solubility and low BBB penetration, higher doses were used, resulting in toxicity [177]. Improved BBB penetration, tissue retention, and decreased efflux with improved survival was noted when panobinostat was delivered by nanotechnology methods [178]. Macrophage exosomes are nanosized vesicles, which can be used as a nanocarrier, for precision guided drug delivery [179]. PPM1D mutation is found in 9–25% of DIPG. This pathogenic gene leads to inactivation of DNA damage response, inducing decreased apoptosis and aggressive proliferation of these tumors. Macrophage exosomes loaded with panobinostat and PPM1D-siRNA were able to knock down the expression of PPM1D in DIPG cells, demonstrating greater killing effect of DIPG tumor cells and prolonging survival of orthotopic DIPG mice compared to controls inhibition [74,75,180]. Ongoing trials are using nanoparticle formulation of panobinostat (MTX110) administered by CED in H3K27M DMG patients (NCT03566199, NCT04264143).

Intranasal delivery of nanoliposomal SN-38, an active metabolite of irinotecan, prolonged survival in a mouse model of DIPG [181]. Based on this promising preclinical data, a phase I clinical trial of CED with irinotecan liposome injection in DIPG patients is ongoing (NCT03086616). Temozolomide, carboplatin, and doxorubicin which have been used systemically in brain tumors, are now being packaged into various forms of nanoparticles and delivered into the CNS tumors with promising results of increased tumor site concentration in preclinical models. These results now provide avenues to be tested in patients [174,182,183,184,185]. Use of gold Np (AuNP) in various cancers is gaining momentum [186]. A nanotechnology model using AuNPs loaded with an arginine-glycine-aspartic acid-like peptide, conjugated with doxorubicin, was used in preclinical models of GBM, which demonstrated lower systemic toxicities and high drug concentration in the tumor. The study demonstrated lower systemic toxicities and high drug concentration in the tumor, achieving an IC50 half that of 40 μg/mL for AuNP alone with a 3.7-fold higher accumulation in tumor cells.

Even though the last decade has witnessed a surge in use of nanotechnology to improve drug delivery of various therapeutics, this method remains challenging in pediatric tumors. To date FDA has approved nearly 20 nanoparticles pediatric cancers, but only a small improvement in survival and reduction of toxicities are seen [187]. Further research should continue to optimize efficacy, safety, and improve pharmacodynamic effects of the nanotechnologies in pediatric brain tumors.

#### 5.4.5. BBB Peptide Shuttles

Specialized to facilitate the transport of therapeutic agents across the barrier, BBB peptide shuttles are yet another expanding area of research in treating brain tumors [188]. These shuttles are classified based on their transport mechanism, ranging from passive diffusion to receptor-mediated transcytosis (RMT).

RMT peptide shuttles mimic natural ligands of specific receptors on the BBB, enabling their transport across the barrier. Examples include those that target the transferrin receptor (TfR), low-density lipoprotein receptor (LDLR), or insulin receptor (InsR). Their transport mechanisms are highly specific which creates significant potential for therapeutics for pediatric populations [189,190]. Transferrin-conjugated carbon dots used to deliver doxorubicin showed that the conjugate had significantly higher uptake and cytotoxicity across multiple GBM cell lines, reducing viability at 10 nM by 14–45% compared to doxorubicin alone [191]. Similar results have been demonstrated with several 1,2,4-triazole derivatives via conjugation with peptide shuttles targeting αvβ6 integrin. More than 50% cytotoxicity against multiple GBM lines was achieved with 50 μg/mL, which was more effective than temozolomide, while also being nontoxic to healthy controls [192].

Drugs or drug delivery systems such as nanoparticles or liposomes can be modified with specific receptor ligands, enabling their internalization via endogenous transport mechanisms. RMT shuttles not only provide precise targeting but also allow the transport of large, polar molecules across the BBB that would otherwise have limited penetration [193]. CD276/B7-H3, highly expressed in medulloblastoma, is a promising target: a recent study demonstrated ligand concentration-dependent uptake of polymeric micelles into MB cell lines [194]. Targeting norepinephrine transporter (hNET) of neuroblastomas, a study confirmed the successful delivery of an ellipticine payload by coating ferritin-based nanovehicles with specific decapeptides, leading to higher levels of both early apoptosis (11% compared to non-coated nanovehicles at 7.3%) and late apoptosis (9.3% compared to 4.4%) [195].

These molecular vectors can be conjugated with a variety of drugs or incorporated with other technologies to enhance delivery and improve therapeutic efficacy while minimizing off-target effects [196,197]. However, more research is necessary to limit enzymatic degradation and avoid toxicological properties, among other shortcomings [198].

## 6. Conclusions

The last two decades have witnessed fervent research, driving the discovery of novel molecular signatures and therapeutic targets in pediatric brain tumors. However, its translation into clinical efficacy has remained markedly disappointing. New drug developments against these formidable neoplasms have remained painstakingly slow, expensive and fraught with regulatory challenges.

Repurposing of previously approved drugs (oncologic and non-oncologic) against pediatric brain tumors, has garnered significant therapeutic optimism, as it bypasses the expensive time-consuming preclinical and rigorous early phase safety studies needed in a new drug development. In parallel, repurposing of drugs using diverse novel drug delivery approaches circumventing the BBB, are now actively profiled to enhance drug concentration in the CNS tumors, mitigating the toxicities associated with systemic drug delivery.

Profiling newer targeted therapy by repurposing PDL-1 and MEK inhibitors (oncologic drugs), m-TOR inhibitors and autophagy inducers (non-oncology drugs) in combination with other standard chemotherapies, could improve therapeutic efficacy. Research should also continue to profile drug delivery to CNS tumors, using novel methods such as CED or FUS, which have shown promise in pediatric neurooncology.

## 7. Future Directions

Future endeavors need to further explore the use of non-oncologic drugs, as they have the unique potential to target multiple unrelated pathways and hit undruggable targets in tumors, not seen with the conventional molecularly targeted therapy. Research efforts should also focus on the use of these repurposed drugs, being optimally and safely redirected into the CNS, using appropriate drug delivery techniques.

Well-orchestrated efforts should continue to hasten and improve the identification of optimal candidate drug, using computational and experimental based approaches. In parallel, rapid development of preclinical models reflecting the molecular profile of tumors of interest, should be pursued. This would facilitate testing of the drug of interest in a timely fashion in vivo for efficacy, CNS penetrance, safety, and FDA approval, prior to it being driven into the clinical arena.

The ultimate success of drug repurposing in this orphan disease will ensue, with a relentless concerted systematic collaborative approach, between scientists, physicians, regulatory bodies, and the pharmaceutical industry. These much-needed efforts, though challenging, are worthwhile, as they usher a new horizon in pediatric CNS cancer therapeutics.

## Figures and Tables

**Table 1:** Repurposed Oncology Drugs.

Drug	Mechanism of Action	Initial FDA Approval	InitialRoute	Repurposed Route	Clinical Trials	Comments
Methotrexate (MTX) PMID: 26346136 PMID: 26255071 PMID: 32330099	Antifolate antimetabolite which inhibits dihydrofolate reductase	Rheumatoid Arthritis and Acute Lymphoblastic Leukemia (ALL)	IV	IVT	NCT05535166	In combination with chemotherapy and +/−irradiation in medulloblastoma (MB)
					NCT06466798	In combination with Nivolumab and Azacitidine (5-AZA) in recurrent CNS malignancies
					NCT02905110	In combination with Etoposide IVT in recurrent posterior fossa tumors
Azacitidine (5-AZA) PMID: 35409174 PMID: 28429280 PMID: 23672687 PMID: 27317342	Inhibits DNA methyltransferase	Myelodysplastic Syndrome	PO/IV/SQ	IV	NCT06466798	In combination with Nivolumab and IVT MTX
Panobinostat PMID: 37827699 PMID: 28915627 PMID: 23054560	Inhibits histone deacetylase	Medulloblastoma	IV	IVT	NCT04315064	Single agent in MB
Sonidegib PMID: 34298664 PMID: 35316996 PMID: 31362788 PMID: 28605510 PMID: 34409296	Smoothened inhibitor	Basal Cell Carcinoma	PO	PO	NCT04402073	Personalized risk adapted therapy in MB
Difluormethylorn-ithine (DFMO) PMID: 25935110 PMID: 33579942 PMID: 30941997 PMID: 12631596 PMID: 11163499	Irreversibly binds to ornithine decarboxylase	Neuroblastoma	PO	PO	NCT04696029	Being evaluated as maintenance therapy in CNS tumors
Prexasertib PMID: 33881209 PMID: 31669383	Selective ATP-competitive small molecule inhibitor of CHK1 and CHK2	Ovarian and Endometrial Cancer	IV	IV	NCT04023669	In Combination with Cyclophosphamide and Gemcitabine in Group 3/4 or SHH MB
Gemcitabine PMID: 38095539 PMID: 29074439 PMID: 35709750 PMID: 39208799 PMID: 31724795 PMID: 32180106	Inhibitor of ribonucleotide reductase	Non-Small Cell Lung Cancer (NSCLC) and Metastatic Breast Cancer	IV	IV	NCT04023669	In Combination with Cyclophosphamide and Prexasertib in G3/4 or SHH MB
					NCT06485908	In combination with Axitinib as metronomic dosing
Etoposide PMID: 32547627 PMID: 38778441 PMID: 37883081 PMID: 9142202 PMID: 27147083 PMID: 23666235 PMID: 30707305 PMID: 22147459	Topoisomerase II inhibitor	Solid Tumors	IV	PO	NCT01356290	Metronomic administration
				IVT	NCT02905110	In combination with MTX IVT in recurrent posterior fossa tumors
Temozolomide (TMZ) PMID: 23666235 PMID: 37649234 PMID: 33844469 PMID: 27006176 PMID: 38596718	Alkylates/methylates DNA	Malignant Brain Tumors	PO	PO	NCT04501718	In combination with Apatinib and Etoposide
					NCT06161974	In combination with Olutasidenib in HGG
					NCT04337177	In combination with PO Irinotecan in MB
Irinotecan PMID: 23459995 PMID: 23630159	Topoisomerase I inhibitor	Colorectal Cancer	IV	PO	NCT04337177	In combination with TMZ in MB
Axitinib PMID: 38778441 PMID: 24967104 PMID: 35008234 PMID: 25213669	Tyrosine Kinase inhibitor (TKI)	Renal Cell Carcinoma	PO	PO	NCT06485908	In combination with metronomic dosing of Etoposide
Apatinib PMID: 33187916 PMID: 37466833 PMID: 34635636 PMID: 37614429 PMID: 30344715	Vascular endothelial growth factor receptor 2 (VEGF-2) inhibitor	Gastric Cancer	PO	PO	NCT04501718	in combination with TMZ and Etoposide
PLX038 PMID: 38898077	Topoisomerase I inhibitor	Colorectal Cancer	IV	IV	NCT06161519	MYCN or MYC amplified CNS tumors
Nivolumab PMID: 30742119 PMID: 32437507 PMID: 35419607 PMID: 38885356 PMID: 35976319 PMID: 30742120	Programmed Death 1 (PD-1) inhibitor	Melanoma	IV	IVT	NCT03173950	Single agent in CNS tumors
				IVT	NCT06466798	In combination with IVT MTX and 5-AZA in recurrent CNS malignancies
Volitinib PMID: 25148209	TKI-mesenchymal epithelial transition factor inhibitor	NSCLC	PO	PO	NCT03598244	Single agent in CNS tumors
Pembrolizumab PMID: 31682550 PMID: 30742122 PMID: 30742119 PMID: 37188783 PMID: 33199490	PD-1 inhibitor	Metastatic NSCLC	IV	IV	NCT02359565	Single agent in HGG, DIPG, ependymoma, MB or hyper-mutated tumors
Bevacizumab PMID: 33007724 PMID: 31701281 PMID: 33796887 PMID: 38733390 PMID: 31252301 PMID: 37883081	VEGF inhibitor	Colorectal Cancer	IV	IV	NCT01356290	Drugs are given on a frequent metronomic schedule with thalidomide, fenofibrate and celecoxib to target proliferating tumor cells and minimize toxicity in recurrent medulloblastoma, ependymoma and ATRT
Cyclophosphamide PMID: 39239066 PMID: 31765733 PMID: 16943538 PMID: 16908838 PMID: 37883081	Nitrogen mustard that alkylates DNA	Lymphoma	IV	PO		
Cytarabine PMID: 28721010 PMID: 24914829 PMID: 38607071 PMID: 27086069 PMID: 22156796	Inhibits DNA polymerase	Leukemia and Lymphoma	IV	PO		
Vorinostat PMID: 34935967 PMID: 22923449 PMID: 34246076 PMID: 34347089	Histone deacetylase inhibitor	Cutaneous T-Cell Lymphoma	PO	PO	NCT00867178	In combination with isotretinoin in children with embryonal brain tumors
Ribociclib PMID: 35709750 PMID: 36318650 PMID: 33547201 PMID: 35611273	Cyclin dependent kinase 4 and 6 (CDK 4/6) inhibitor	Breast Cancer	PO	PO	NCT05843253	In combination with Everolimus in HGG and DIPG
Everolimus PMID: 33547201 PMID: 37978951 PMID: 34551970 PMID: 35611273 PMID: 33140540	Mammalian target of rapamycin (mTOR) inhibition	Gastric and Lung Neuroendocrine Tumors	PO	PO	NCT05843253	In combination with Ribociclib in LGG
					NCT04485559	In combination with Trametinib in LGG
Trametinib PMID: 37733309 PMID: 34838156 PMID: 36375115 PMID: 31881853 PMID: 37643378	Mitogen activated extracellular signal regulated kinase (MEK) 1–2 inhibitor	Melanoma	PO	PO	NCT04485559	In combination with Everolimus in LGG
Olutasidenib PMID: 31971798	Isocitrate dehydrogenase-1 (IDH-1) inhibitor	Acute Myeloid Leukemia	PO	PO	NCT06161974	In combination with TMZ in HGG and DIPG
Mirdametinib PMID: 38714355 PMID: 36451044	Mitogen activated extracellular signal regulated kinase (MEK) 1–2 inhibitor	Plexiform Neurofibroma	PO	PO	NCT04923126	Single agent in LGG
Selumetinib PMID: 31151904 PMID: 33631016 PMID: 31151905 PMID: 35363510	Mitogen activated extracellular signal regulated kinase (MEK) 1–2 inhibitor	Plexiform Neurofibroma	PO	PO	NCT03871257	Single agent in LGG
					NCT04166409	
					NCT04576117	In combination with Vinblastine in progressive LGG
Vinblastine PMID: 22393086 PMID: 27573663 PMID: 33235152 PMID: 32666050 PMID: 27037940 PMID: 30848061	Microtubule inhibitor	ALL	IV	IV	NCT06381570	In combination with Tovorafenib with RAF altered LGG
					NCT04576117	In combination with Selumetinib in progressive LGG

End Note notations indicate studies that have been published and completed and clinical trial identifiers are trials that are ongoing or not yet published. MTX: Methotrexate, IVT: Intraventricular, ALL: Acute lymphoblastic leukemia, 5-AZA: Azacitidine, DFMO: Difluormethylorn-ithine, CNS: Central Nervous System, MB: Medulloblastoma, NSCLC: Non-small cell lung cancer, HGG: High-grade glioma, DIPG: Diffuse intrinsic pontine glioma, TKI: Tyrosine kinase inhibitor, VEGF: Vascular endothelial growth factor, ATRT: Atypical teratoid rhabdoid tumor, CDK: Cyclin dependent kinase, mTOR: Mammalian target of rapamycin, MEK: Mitogen activated extracellular signal regulated kinase, LGG: low-grade glioma.

**Table 2:** Non-Oncologic Repurposed Drugs.

Drug	Mechanism of Action in Oncology	Initial FDA Approval	InitialRoute	Repurposed Route	Completed and Active Clinical Trials	Comments
Valproic Acid (VPA) PMID: 26194676 PMID: 21115653 PMID: 37114548 PMID: 32537176 PMID: 32285998	Type I HDAC inhibitor. Enhances recruitment of tumor suppressor factors, inducing apoptosis, differentiation, inhibition of cell cycle, targets MAPK, β-catenin, GSK-3 signaling pathway, enhances radiation	Epilepsy	PO	PO	NCT02265770	In combination with chemotherapy in those with ependymoma that cannot receive irradiation
					NCT00107458	Single agent study
					NCT00302159	In combination with TMZ and irradiation in glioblastoma
					NCT00879437	In combination with Bevacizumab and irradiation in HGG and DIPG
					NCT03243461	In combination with TMZ and irradiation in HGG
Levetiracetam PMID: 25975354 PMID: 25975354 PMID: 34982371	Inhibits cell growth and proliferation, increases GBM cells sensitivity to TMZ and RT, Inhibits HDAC, downregulates MGMT	Epilepsy	PO/IV			
Aprepitant PMID: 34377985	NK-1 receptor inhibitor	Chemotherapy induced nausea-vomiting (CINV)	IV			
Sirolimus/ Temsirolimus PMID 9721431 PMID: 18215105 PMID:32843426 PMID: 32843426	mTOR inhibitor which inhibits the progression at G1 and S phase of cell cycle	Prophylaxis of organ rejection in renal transplants	PO	PO/IV/Intra-arterial cerebral infusion	NCT02420613	Temsrolimus in combination with vorinostat and irradiation in DIPG
					NCT02574728	Sirolimus with metronomic chemotherapy in CNS tumors
					NCT00329719	Temsirolimus in combination with sorafenib in patients with recurrent glioblastoma
					NCT00016328	Temsirolimus in recurrent glioblastoma
					NCT05773326	Temsirolimus intra-arterial cerebral infusion in HGG
					NCT02238496	Temsirolimus and perifosine in recurrent glioma
					NCT00316849	Temsirolimus, TMZ and irradiation in glioblastoma
					NCT03463265	Nab-sirolimus in combination with combination chemotherapy in recurrent glioblastoma
					NCT00672243	Single agent sirolimus in recurrent glioma multiforme
					NCT00112736	Temsirolimus and Erlotinib in recurrent glioma
Celecoxib PMID: 24123865 PMID: 24692119 PMID: 30073924	Cyclooxygenase-2 (COX-2) inhibitor	Osteoarthritis and rheumatoid arthritis	PO	PO	NCT01356290	Drugs are given on a frequent metronomic schedule to target proliferating tumor cells and minimize toxicity in recurrent medulloblastoma, ependymoma and ATRT
					NCT02574728	In combination with metronomic chemotherapy in CNS tumors
					NCT0047281	In combination with thalidomide and chemotherapy in recurrent glioma
					NCT00504660	In combination with 6-TG, capecitabine, CCNU or TMZ in anaplastic glioma
Chloroquine/ Hydroxychloroquine PMID: 33413069 PMID: 32866424 PMID: 28094001	Break/discontinue autophagosome-lysosome fusions, thereby modulating autophagy, inducing apoptosis, eliminating cancer stem cells.	Antimalarial	PO	PO	NCT00486603	In combination with irradiation and TMZ in glioblastoma
					NCT04201457	In combination with dabrafenib and trametinib in LGG and HGG
					NCT02496741	In combination with metformin in IDH mutated solid tumors
Mebendazole PMID: 33506200 PMID: 36674870	Inhibits polymerization of b-tubulin into microtubules thereby suppressing DNA synthesis, cell migration and invasion, inhibiting angiogenesis, G2/M cell cycle arrest.	Anti-helminthic	PO	PO	NCT02644291	Single agent in recurrent CNS tumors
					NCT06485908	Single agent in pediatric glioma
					NCT01729260	In combination with TMZ in HGG
Metformin PMID: 29058720 PIMD: 33869859 PMID: 36151773 PMID 34613815	It inhibits metabolic reprogramming and interplay between the metabolic and epigenetic landscapes, now widely recognized as oncorequisite factors in the pediatric hindbrain	Diabetes	PO	PO	NCT04945148	In combination with irradiation and TMZ
					NCT02496741	In combination with chloroquine in IDH mutated solid tumors
					NCT01528046	In combination with vincristine, irinotecan and TMZ in relapsed solid tumors
					NCT01430351	In combination with TMZ, memantine, and mefloquine in glioblastoma
Memantine PMID: 30359477 PMID: 38225589	Inhibits proliferation and induces autophagy mediated by NMDAR1 and prevents acute toxicities of radiation	Alzheimer’s disease Dementia	PO	PO	NCT01430351	In combination with TMZ, metformin, and mefloquine in glioblastoma
Disulfiram PMID: 31084595 PMID: 26966095 PMID: 30771200	It induces oxidative stress by the generation of ROS, inhibition of superoxide dismutase activity, suppression of the ubiquitin-proteasome system, and activation of MAPK pathway. Also inhibits DNA methyltransferase, and MGMT.	Alcoholism	PO	PO	NCT02678975	In combination with chemotherapy and copper in recurrent glioblastoma
					NCT01907165	In combination with TMZ with or without copper post irradiation in glioblastoma
					NCT02770378	In combination with TMZ and 8 repurposed drugs in a metronomic regimen
Fluoxetine/Sertraline PMID: 36543105 PMID: 36113478 PMID: 28500556	Selective serotonin reuptake inhibitor (SSRI). Inhibiting SMPD1 mediated sphingomyelin metabolism and preventing epidermal growth factor receptor variant III (EGFRvIII) signaling.	Depression	PO	PO	NCT05634707	In combination with TMZ post biopsy as maintenance therapy in glioma
					NCT02770378	In combination with TMZ and 8 repurposed drugs in a metronomic regimen
Thalidomide PMID: 23931282 PMID: 34165402 PMID: 37883081 PMID:18760193 PMID: 17031553 PMID: 22147459	Anti-angiogenic properties targeting FGF2 and IGF1. As well as COX-2 inhibitor.	Leprosy and multiple myeloma	PO	PO	NCT01356290	Combination of medications in a metronomic regimen (MEMMAT)
					NCT00098865	In combination with TMZ in relapsed CNS tumors
					NCT00006358	
					NCT00039468	In combination with Irinotecan in glioblastoma
					NCT00251797	
					NCT00179881	In combination with Carboplatin in brainstem glioma
					NCT00047294	In combination with TMZ and Celecoxib post irradiation with glioblastoma

End Note notations indicate studies that have been published and completed and clinical trial identifiers are trials that are ongoing or not yet published. HDAC: Histone deacetylase, MAPK: Mitogen-activated protein kinase, GSK-3: Glycogen synthase kinase-3, GBM: Glioblastoma multiforme, TMZ: Temozolomide, RT: Radiotherapy, MGMT: O 6 -methylguanine-DNA methyltransferase, NK-1 receptor: neurokinin 1 receptor, CUSP9v3 trial: Coordinated Undermining of Survival Paths combining 9 repurposed non-oncological drugs with metronomic temozolomide-version 3, mTOR: mammalian target of rapamycin, PI3K/AKT: Phosphatidylinositol-3-kinase (PI3K), BRAF: protein encoded by BRAF gene, FKBP12: member of a family of FK506 -binding proteins (FKBPs), PGE2: Prostaglandin E2, NF-κB: Nuclear factor kappa-light-chain-enhancer of activated B cells, STAT3: Signal transducer and activator of transcription 3, MMP: Matrix metalloproteinase, HGG: High-grade glioma, EPN: ependymoma, MB: medulloblastoma, LGG: Low-grade glioma, ROS: Reactive oxygen species, TME: Tumor microenvironment, EZHIP: Enhancer of Zeste Homologs Inhibitory Protein, H3K27Mme3: Tri-methylation of the 27th lysine residue on histone H3, TCA: Tricarboxylic acid cycle, PFA: Posterior Fossa Group A ependymoma, NMDAR1: N-methyl-D-aspartate receptor 1, SMPD1: sphingomyelin phosphodiesterase-1, VEGF: Vascular endothelial growth factor, GSC: Glioma stem cell, SLE: Systemic lupus erythematosus, MDS: Myelodysplastic syndrome, FGF2: Fibroblast growth factor 2, IGF1: Insulin Growth Factor 1.

**Table 3:** Outcomes for repurposed oncologic drugs.

Drug	CNS Tumor Type	Publication Results
Methotrexate (MTX) PMID: 26346136 PMID: 26255071 PMID: 32330099	Medulloblastoma	IVT Methotrexate is both safe and feasible for children with medulloblastoma. Higher doses were associated with better survival.
	Ventricular CNS tumors	IVT Methotrexate demonstrated efficacy in patients with medulloblastoma with anti-tumoral effect.
	Medulloblastoma	IVT Methotrexate in combination with chemotherapy improved survival in patients with infant sonic hedgehog medulloblastoma.
Azacitidine (5-AZA) PMID: 35409174 PMID: 28429280 PMID: 23672687 PMID: 27317342	Medulloblastoma	Epigenetic regulators offer improved survival in both pre-clinical studies using cell lines as well as mice receiving multimodal treatment.
Panobinostat PMID: 37827699 PMID: 28915627 PMID: 23054560	Medulloblastoma	Panobinostat suppressed tumor growth in mouse models as well as cell lines in pre-clinical data.
Sonidegib PMID: 34298664 PMID: 35316996 PMID: 31362788 PMID: 28605510 PMID: 34409296	Medulloblastoma	Sonidegib is well tolerated and demonstrate anti-tumor activity in patients with Sonic Hedge Hog mutant medulloblastoma. It has also demonstrated prolonged progression free survival in case reports.
Difluormethylorn-ithine (DFMO) PMID: 25935110 PMID: 33579942 PMID: 30941997 PMID: 12631596 PMID: 11163499	Glioblastoma	DFMO has demonstrated efficacy in glioblastoma cell lines in combination with chemotherapy and radiation.
	Diffuse Midline Glioma	It has demonstrated efficacy as a single agent in diffuse midline glioma of the pons.
Prexasertib PMID: 33881209 PMID: 31669383	Ependymoma	Prexasertib led to stable disease in patients with ependymoma in a previous clinical trial.
Gemcitabine PMID: 38095539 PMID: 29074439 PMID: 35709750 PMID: 39208799 PMID: 31724795 PMID: 32180106	Multiple CNS Tumors	Gemcitabine is anti-tumorigenic in combination with other chemotherapeutic agents in multiple CNS tumor types including diffuse midline glioma, glioblastoma, medulloblastoma, atypical teratoid rhabdoid tumor, and intracranial germ cell tumor.
Etoposide PMID: 32547627 PMID: 38778441 PMID: 37883081 PMID: 9142202 PMID: 27147083 PMID: 23666235 PMID: 30707305 PMID: 22147459	Recurrent CNS Tumors	Etoposide has shown activity in recurrent brain tumors in combination with other chemotherapeutic agents, both as a traditional chemotherapeutic agent and when utilized in a metronomic, anti-angiogenic fashion. It is safe and feasible when used as an intraventricular agent in recurrent brain tumors.
Temozolomide (TMZ) PMID: 23666235 PMID: 37649234 PMID: 33844469 PMID: 27006176 PMID: 38596718	Glial Tumors	Temozolomide has demonstrated efficacy in glial tumors, but some tumors demonstrate resistance, therefore limiting its use in certain populations.
	Medulloblastoma	TMZ has been used effectively to treat recurrent medulloblastoma in combination with other agents.
Irinotecan PMID: 23459995 PMID: 23630159	Recurrent CNS Tumors	Both oral and IV irinotecan have demonstrated improved survival in patients with recurrent brain tumors when used in combination with other chemotherapeutic agents.
Axitinib PMID: 38778441 PMID: 35008234 PMID: 25213669	Recurrent CNS Tumors and Glioblastoma	Axitinib has shown efficacy in combination therapy in heavily treated patients with CNS tumors. Single agent activity has been demonstrated in glioblastoma cell lines.
Apatinib PMID: 33187916 PMID: 37466833 PMID: 34635636 PMID: 37614429 PMID: 30344715	High-Grade Glioma	Apatinib inhibits cell growth and promotes apoptosis in glioma cell lines and prolongs survival in small studies of patients with recurrent high-grade glioma.
PLX038 PMID: 38898077	Glioblastoma	PLX038 penetrates the blood brain barrier and improves survival in glioblastoma mice models.
Nivolumab PMID: 30742119 PMID: 32437507 PMID: 35419607 PMID: 38885356 PMID: 35976319 PMID: 30742120	Glioblastoma	Nivolumab demonstrates clinical response in glioblastoma, with one study revealing efficacy among glioblastoma with specific genetic signatures. Nivolumab crosses the blood brain barrier and upregulates checkpoint pathways.
Volitinib PMID: 25148209	Multiple CNS Tumors	Volitinib has demonstrated dose dependent tumor growth inhibition in cancer studies in mice and is being actively studied in early phase clinical trials.
Pembrolizumab PMID: 31682550 PMID: 30742122 PMID: 30742119 PMID: 37188783 PMID: 33199490	Glioblastoma	Pembrolizumab has demonstrated clinical benefit in patients with high microsatellite instability/tumors with DNA mismatch repair deficiency. When used as a neoadjuvant there is survival benefit in recurrent glioblastoma.
Bevacizumab PMID: 33007724 PMID: 31701281 PMID: 33796887 PMID: 38733390 PMID: 31252301 PMID: 37883081	High-Grade Glioma	Bevacizumab has demonstrated efficacy in high grade glioma, both alone and in combination with chemotherapy and irradiation.
	Low-Grade Glioma	It is also effective in optic pathway glioma, with visual preservation and improvement noted in multiple case studies/series.
	Medulloblastoma	It has also shown improved survival in recurrent medulloblastoma, both as conventional chemotherapy and when used in a metronomic regimen.
Cyclophosphamide PMID: 39239066 PMID: 31765733 PMID: 16943538 PMID: 16908838 PMID: 37883081	High-Grade Glioma	Cyclophosphamide acts synergistically with other chemotherapeutic agents in recurrent malignant glioma.
	Medulloblastoma	It has been used in combination with multimodal therapy in medulloblastoma. Additionally, when used as part of metronomic therapy it has a sustained survival benefit in recurrent medulloblastoma.
Cytarabine PMID: 28721010 PMID: 24914829 PMID: 38607071 PMID: 27086069 PMID: 22156796	High-Grade Glioma	Cytarabine has shown activity on leptomeningeal metastasis and primary tumors in high-grade glioma. It has also been studied in xenografts as a cytarabine loaded vesicular phospholipid gel.
	Medulloblastoma	It has also been used intrathecally with metronomic therapy in medulloblastoma.
Vorinostat PMID: 34935967 PMID: 22923449 PMID: 34246076 PMID: 34347089	Diffuse Midline Glioma	Vorinostat has been incorporated into traditional chemotherapy regimens safely and feasibly. It has also demonstrated synergy in multiple tumor types including diffuse midline glioma of the pons. When used in combination with irradiation in diffuse midline glioma of the pons it is well tolerated but did not confer a survival benefit.
Ribociclib PMID: 35709750 PMID: 36318650 PMID: 33547201 PMID: 35611273	Medulloblastoma	Ribociclib in combination with chemotherapy showed a decrease in tumor activity in both mouse and human patient derived orthotopic xenograft models in medulloblastoma.
Everolimus PMID: 33547201 PMID: 37978951 PMID: 34551970 PMID: 35611273 PMID: 33140540	Low-Grade Glioma	Everolimus is well tolerated in children with recurrent brain tumors, including certain genetically defined low-grade glioma. Its use resulted in a partial response in previously treated population.
	High-Grade Glioma	Everolimus is safe for patients with diffuse midline glioma of the pons and high-grade glioma in combination with other targeted therapies.
Trametinib PMID: 37733309 PMID: 34838156 PMID: 36375115 PMID: 31881853 PMID: 37643378	Low-Grade Glioma	Trametinib plus Dabrafenib treatment resulted in greater response rate, longer progression free survival and improved safety profile in patients with a low-grade glioma with BRAFV600e mutations.
	High-Grade Glioma	In high-grade glioma with this mutation it also demonstrated clinically meaningful activity.
	Plexiform Neurofibroma	Trametinib is being studied in patients with plexiform neurofibromas and neurofibromatosis type 1.
Olutasidenib PMID: 31971798	High-Grade Glioma	Olutasidenib is a potent mutant selective IDH1 inhibitor which has demonstrated anti-tumor activity in xenograft tumor models.
Mirdametinib PMID: 38714355 PMID: 36451044	High-Grade Glioma	Midametinib has demonstrated synergistic activity with irradiation in neurofibromatosis type 1 deficient glioma models.
Selumetinib PMID: 31151904 PMID: 33631016 PMID: 31151905 PMID: 35363510	Low-Grade Glioma	Selumetinib is effective in patients with BRAF aberrant recurrent, progressive, or refractory low-grade glioma, including those with optic pathway glioma; demonstrating prolonged disease stability.
Vinblastine PMID: 22393086 PMID: 27573663 PMID: 33235152 PMID: 32666050 PMID: 27037940 PMID: 30848061	Low-Grade Glioma	Vinblastine is comparable to first line chemotherapy in pediatric refractory low-grade glioma. Its use in a metronomic monotherapy regimen in combination with Bevacizumab and Irinotecan prevents early relapse.
	Medulloblastoma	It also demonstrated equivalent antitumor activity as vincristine in medulloblastoma in a case series.

**Table 4:** Outcomes for repurposed non-oncologic drugs.

Drug		
Valproic Acid (VPA) PMID: 26194676 PMID: 21115653 PMID: 37114548 PMID: 32537176 PMID: 32285998	Glioblastoma	The addition of valproic acid to chemo-irradiation results in improved outcomes in comparison to historical data in small cohorts.
	High-Grade Glioma	Valproic acid was well tolerated but did not improve outcomes in pediatric high-grade glioma and diffuse midline glioma of the pons.
Levetiracetam PMID: 25975354 PMID: 34982371	Glioblastoma	Levetiracetam improved survival in patients receiving chemo-irradiation in small cohorts. It has demonstrated efficacy in specific molecular subtypes of glioblastoma.
Aprepitant PMID: 34377985	Glioblastoma	Aprepitant has been administered safely in combination with other repurposed drugs, and is being studied in higher phase studies.
Sirolimus/ Temsirolimus PMID: 18215105 PMID: 32843426	Glioblastoma	Sirolimus has demonstrated anti-tumor activity when used alone or in combination in PTEN-deficient glioblastoma.
	BRAF mutated CNS Tumors	Co-targeting the MAPK and mTOR pathway in BRAF mutated CNS Tumors led to improved radiographic and clinical response in early phase clinical trials.
Celecoxib PMID: 24123865 PMID: 24692119 PMID: 30073924	Multiple CNS Tumors	Celecoxib has been used as part of a metronomic regimen with both partial response and stable disease demonstrated in different tumor types.
Chloroquine/ Hydroxychloroquine PMID: 33413069 PMID: 32866424 PMID: 28094001	Glioblastoma	Chloroquine results in autophagy and reduction in tumor size in glioblastoma xenografts and been used safely in combination with irradiation in humans.
	Low-Grade Glioma	Chloroquine overcomes resistance mechanisms in low grade glioma.
Mebendazole PMID: 33506200 PMID: 36674870	High-Grade Glioma	Mebendazole demonstrate long term safety and acceptable toxicity but need to be studied to a greater degree to determine efficacy.
Metformin PMID: 29058720 PMID: 33869859 PMID: 36151773	High-Grade Glioma	Metformin demonstrates improved overall survival and progression free survival in therapy with temozolomide.
Memantine PMID: 30359477 PMID: 38225589	Glioblastoma	Memantine can safely be combined with other repurposed drugs post chemo-irradiation in glioblastoma, with efficacy data being studied. It is also being actively studied in combination of chemo-irradiation in clincal trials.
Disulfiram PMID: 31084595 PMID: 26966095 PMID: 30771200	Glioblastoma	Disulfiram was limited in combination with TMZ due to the MTD, limiting proteasome inhibition on peripheral blood cells.
Fluoxetine/Sertraline PMID: 36543105 PMID: 36113478 PMID: 28500556	Glioma	Tricyclic antidepressants in combination with antiangiogenic therapy are efficacious in genetically engineered glioma models as well as mouse models. Inclusion of immune-checkpoint blockade improves survival in patients with glioblastoma.
Thalidomide PMID: 23931282 PMID: 34165402 PMID: 37883081 PMID:18760193 PMID: 17031553 PMID: 22147459	Medulloblastoma	Thalidomide is effective in medulloblastoma when used in combination with other agents in a metronomic fashion.
	Brainstem CNS Tumors	Metronomic treatment with carboplatin, vincristine, Fluvastatin and thalidomide significantly increase survival of pediatric brain stem tumor patients in small cohorts.
	Glioblastoma	The addition of thalidomide to irradiation in high-grade glioma did not improve time to progression or time to death compared to historical cohorts.
